# Drug-Induced Parkinsonism: Too Many Cooks in the Kitchen

**DOI:** 10.7759/cureus.44896

**Published:** 2023-09-08

**Authors:** Mark Liotta, Harrison Bell, Anh-Thu Vu, Michael Stillman

**Affiliations:** 1 Internal Medicine, Thomas Jefferson University Hospital, Philadelphia, USA; 2 Neurology, Thomas Jefferson University Hospital, Philadelphia, USA

**Keywords:** drug-induced parkinsonism, antidopaminergic, iatrogenic complication, datscan, antipsychotics, parkinson' s disease

## Abstract

Drug-induced parkinsonism (DIP) is a condition characterized by the development of parkinsonian symptoms as a result of medication use. It is often misdiagnosed and can be challenging to differentiate from Parkinson's disease (PD). In this case presentation, we describe the clinical course of a 64-year-old male who presented with parkinsonian symptoms while using atypical antipsychotics, which was originally misdiagnosed as PD.

The case highlights the importance of recognizing the potential iatrogenic effects of medications with antidopaminergic properties, such as antipsychotics and antiepileptic drugs, which are common culprits in causing DIP. We discuss DIP management, long-term impacts, and differentiating DIP from PD through clinical findings and imaging, emphasizing the utility of the (123)I-ioflupane single-photon emission computerized tomography (SPECT) scan in aiding diagnosis.

This case serves as a reminder to healthcare providers to remain vigilant in monitoring patients for adverse effects, polypharmacy, and harmful medication interactions.

## Introduction

Parkinsonism is a syndrome with symptoms including bradykinesia, resting tremor, and/or rigidity due to impairment of the cortico-basal ganglia-thalamo-cortical loop. Parkinson's disease (PD) is the most common etiology of parkinsonism, with more than 1.04 million individuals diagnosed [1]. With an aging population, this number is expected to rise, resulting in an estimated economic burden of $73 billion by 2032. Patients with PD also experience an overall poorer quality of life with reduced mobility and increased rates of depression and cognitive impairment, making the completion of activities of daily living challenging [2].

After PD, drug-induced parkinsonism (DIP) is the second most common etiology of parkinsonism, with prevalence rates of 0.09% to 2.7%, and is often misdiagnosed [3]. Understanding the inciting factors, and clinical and imaging features for diagnosis and potential complications of DIP is therefore critical to the early and appropriate identification and management of these patients to improve their overall quality of life. Here we present a case in which a patient developed significant parkinsonian symptoms after initiating and altering antidopaminergic atypical antipsychotic medications.

## Case presentation

A 64-year-old male with a medical history of bipolar disorder and anxiety presented to his primary care provider’s (PCP’s) office for a new patient visit. His medical history was significant for bipolar disorder, hypertension, hyperlipidemia, obstructive sleep apnea, insomnia, and gastroesophageal reflux disease. His medications included cariprazine 3mg daily, lisinopril 20mg daily, chlorthalidone 25mg daily, atorvastatin 20mg daily, lorazepam 1mg nightly as needed for insomnia, and pantoprazole 40mg daily.

For the past six months, he had experienced memory and concentration difficulties, fatigue, tremors, and a shuffling gait. His psychiatrist had been prescribing divalproex 2,500mg daily for many years with good control of his bipolar disorder, but after a breakthrough manic episode three years before presentation, he had transitioned the patient to aripiprazole 5mg daily. When the patient first complained of fatigue and diminished cognitive function, his psychiatrist stopped aripiprazole after three months of use and resumed treatment with divalproex for six months leading up to his PCP appointment. One week before the patient’s primary care appointment, however, he had been taken off divalproex and offered a trial of cariprazine 3mg daily, an atypical antipsychotic, due to concerns that his symptoms were due to divalproex.

Given his constellation of symptoms, the patient went for a general neurological consultation and was noted to have masked facies, left worse than right global bradykinesia, and a fine, postural, right upper extremity tremor. He also endorsed unsteadiness, stiffness, worsened insomnia, constipation, urinary frequency, and mild cognitive deficits, including executive dysfunction and visuospatial as well. He denied any dystonia, hallucinations, or orthostasis.

Initial workup, including thyroid stimulating hormone, vitamin B12, a complete blood count, and a comprehensive metabolic panel, were all within normal limits. An MRI with and without contrast identified some cerebral volume loss and a hypoplastic left vertebral artery, which were unchanged from prior studies. There was no evidence of acute infarction, cerebral edema, mass effect, or intracranial mass to explain his current presentation. Neuropsychological testing diagnosed the patient with a mild neurocognitive disorder.

**Figure 1:**
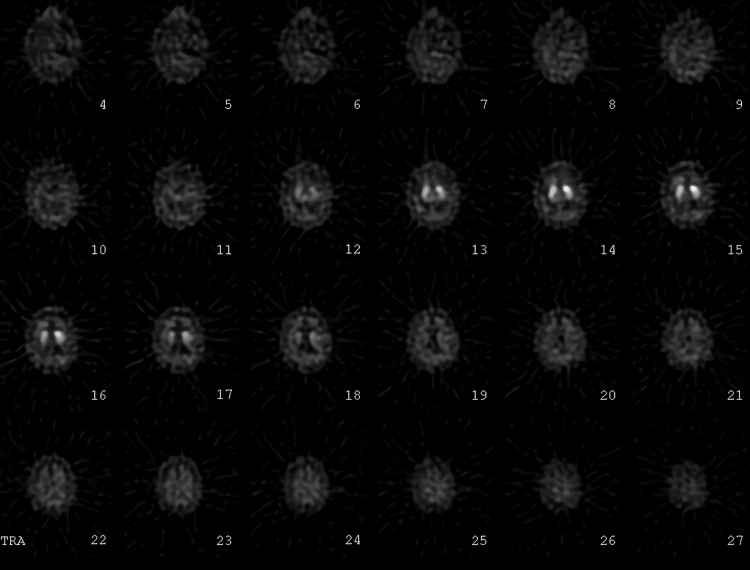
The (123)I-ioflupane SPECT scan with bilateral presynaptic striatal dopaminergic deficits, right worse than left seen in images 13–17 SPECT: single-photon emission computerized tomography

A (123)I-ioflupane single-photon emission computerized tomography (SPECT) scan demonstrated bilateral presynaptic striatal dopaminergic deficits, the right worse than the left (Figure 1), and carbidopa-levodopa 25mg-100mg twice a day was initiated and up-titrated for presumed Parkinson’s disease (PD).

During his neuropsychological evaluation, he was diagnosed with mild cognitive impairment. Additional provider visits noted that despite carbidopa-levodopa use for three months, he experienced the onset of drooling, constipation, and restless legs, and during an emergency department visit for a fall, he was noted to have marked parkinsonism.

He was referred to a movement disorder specialist, who confirmed that the symptoms were consistent with parkinsonism, although his recent use of dopaminergic antagonists made differentiation between idiopathic PD and DIP challenging. His symptoms initially may have been due to the use of aripiprazole or underlying neurodegenerative parkinsonism, but his rapid Parkinsonian symptom progression was attributed to the new initiation of cariprazine. During that evaluation, his Movement Disorder Society-Sponsored Revision of the Unified Parkinson's Disease Rating Scale III (MDS-UPDRS) score was 34 and he received points primarily for facial expressions, speech, gait, rigidity, and fine motor movements.

Carbidopa-levodopa was discontinued, and the patient was transitioned from cariprazine back to divalproex with the help of psychiatry. Within weeks, his gait and constipation had improved, and he no longer experienced falls. At a four-month follow-up exam, Parkinsonian symptoms including stiffness, gait unsteadiness, arm swing and rigidity, and bradykinesia had all resolved or markedly improved. His only symptoms that persisted included a decreased blink rate, restless legs, and a slowed gait without shuffling.

## Discussion

Parkinsonism is a syndrome with symptoms that include bradykinesia, resting tremor, postural instability, and/or rigidity due to dysregulation of the extrapyramidal system. Drug-induced parkinsonism is the second most common etiology of parkinsonism in the elderly after PD and is more common in females. Drug-induced parkinsonism is often misdiagnosed, so determining its incidence and prevalence is difficult. However, prevalence ranges from 0.09% to 2.7%, and up to 6.8% of patients previously diagnosed with PD are eventually determined to have DIP [3].

Medications with antidopaminergic effects are some of the most common causative agents for DIP. These include typical antipsychotics, atypical antipsychotics (aripiprazole, cariprazine), gastrointestinal motility drugs and antiemetics (metoclopramide), some calcium channel blockers, amiodarone, lithium, and antiepileptic drugs such as divalproex (independent from elevated blood ammonia levels) [3,4]. The onset of parkinsonism is typically days to weeks after starting the medication, though the condition may also be precipitated by dose increases [3,5]. The reported incidence of cariprazine-induced DIP is between 8.3% and 15.4% [6].

To be diagnosed with DIP, a patient must have symptoms of parkinsonism during medication use without a history of similar symptoms. Careful history-taking is thus imperative. A (123)I-ioflupane SPECT scan can help differentiate between PD and DIP; however, this is typically reserved for cases of persistent symptoms after medication cessation. For those with neurodegenerative parkinsonism, scans will show reduced radiotracer uptake compared to complete uptake in DIP. Reduced uptake can help identify preliminary stages of PD, as symptom onset typically presents after losing 60%-80% of dopaminergic neurons [1]. Medications such as haloperidol, bupropion, fentanyl, cocaine, methamphetamine, and methylphenidate can interfere with these scans by decreasing striatal binding of (123)I-ioflupane and creating false positive results. It is recommended that a washout period take place prior to a SPECT scan for patients on potentially interfering medications [7].

Clinically, it is difficult to assess the differences between DIP and PD. The symmetry and type of tremor are not generally useful in distinguishing the two. However, DIP patients exhibit more prominent bradykinesia and rigidity, which are generally bilateral and symmetric. Smaller studies have indicated that up to 30%-50% of DIP patients have asymmetric symptoms, which would be more supportive of a PD diagnosis [3]. For equivocal clinical and imaging findings, skin biopsies to detect phosphorylated α-synuclein deposits can help diagnose PD, as such abnormalities are not seen in those with DIP [8].

Cessation of the potentially offending agent and/or switching to an alternative medication with fewer extrapyramidal effects are the appropriate first steps for the treatment of suspected DIP. Parkinsonian symptoms and findings typically improve within weeks to months; however, 10%-50% of patients can have persistence and/or progression of parkinsonism [3]. This may indicate either permanent damage due to antidopaminergic agents or exposed pre-clinical PD exacerbated by medications. Permanent damage from antidopaminergic agents is believed to be due to inhibited mitochondrial respiratory function, ultimately leading to irreversible cell death and dopamine pathway deficits [9]. Exacerbated PD may be due to existing dopaminergic neuron damage and increased α-synuclein deposition, with symptoms reaching clinical levels once dopamine blockers are introduced. The most common medications associated with PD after DIP include calcium channel blockers, antidepressants, and antihistamines. Drug-induced parkinsonism has also been shown to shorten the onset of PD by approximately one year, further supporting this theory.

## Conclusions

In this case, the patient experienced new-onset parkinsonism while using aripiprazole, which then worsened after the initiation of cariprazine. Cessation of treatment with atypical agents and resumption of divalproex resulted in marked symptomatic improvement after a three-month follow-up, yet the patient did have mild residual parkinsonism. Prescribers must always be aware of potential iatrogenic effects and remain vigilant in monitoring for adverse effects, polypharmacy, and harmful medication interactions. Cessation of treatment with atypical antipsychotics should be performed closely with the help of psychiatry to prevent precipitating psychiatric symptoms. As the patient has a dopaminergic deficit observed on a SPECT scan, his persistent symptoms may be due to either a preclinical stage of PD, residual damage to dopamine receptors, continued use of divalproex, or a combination of etiologies. Close follow-up will be critical to continue monitoring symptoms and providing early intervention if PD develops.
